# 3D aerogels from hybrid 2D materials: ultralight and flexible superinsulators

**DOI:** 10.1093/nsr/nwad196

**Published:** 2023-07-14

**Authors:** Stefano Ippolito, Yury Gogotsi

**Affiliations:** A.J. Drexel Nanomaterials Institute and Department of Materials Science & Engineering, Drexel University, USA; A.J. Drexel Nanomaterials Institute and Department of Materials Science & Engineering, Drexel University, USA

Advances in research and technology to boost human space exploration proceed at a brisk pace, although a major obstacle remains: the cold and empty outer space. Can we capitalize on the unique properties of specific materials and structures to address this problem? Considered among the best insulating materials available, aerogels have become prime candidates in this field owing to their ultralight weight, large surface area, deformability, excellent fire and corrosion resistance, as well as low thermal conductivity [1]. However, they suffer from extreme fragility due to ultralow density and high crystallinity [2]. Multiple attempts have been made to optimize the aerogel design. 2D materials with large subnanometer-thin flakes like graphene [3,4] and boron nitride (BN) [5] have shown promise. However, the studies to date have succeeded in maximizing either the thermal or the mechanical properties, but not both at the same time.

**Figure 1.**
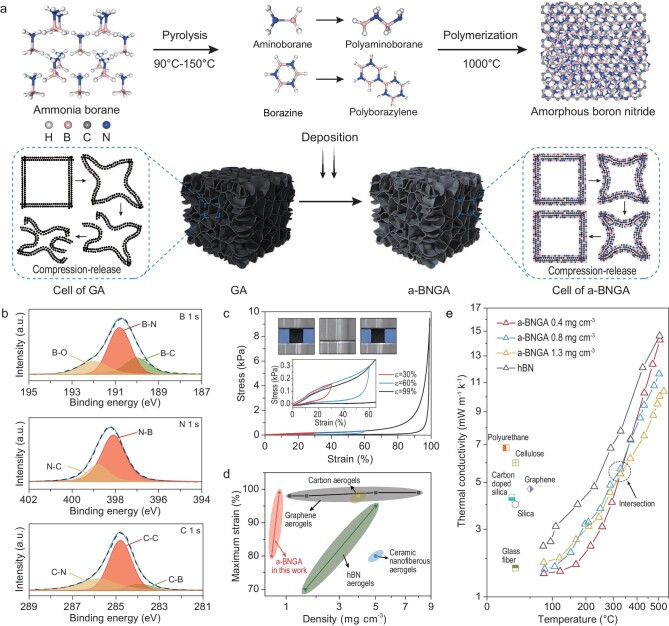
Production of ultralight, flexible and superinsulating aerogels. (a) Illustration of the synthesis protocol for a-BNGA involving the thermal decomposition and polymerization of ammonia borane on graphene aerogels. (b) High-resolution X-ray photoelectron spectroscopy (XPS) spectra of B 1s, N 1s and C 1s for a-BNGA. (c) Uniaxial compression of a-BNGA with repeatable strain up to 99%. (d) Comparison of the maximum strain and density between a-BNGA and conventional inorganic aerogels. (e) Comparison of thermal conductivity in vacuum between a-BNGA and other aerogels reported in the literature. Reprinted with permission from Ref. [6].

Writing in *National Science Review*, Yu *et al*. overcome this critical issue by using an innovative approach featuring hybrid 3D aerogels made of chemically bonded 2D inorganic materials, namely graphene and amorphous boron nitride (a-BNGA) [6]. They developed a method to improve the mechanical and thermal properties simultaneously, taking advantage of a synthesis protocol in which ammonia borane (BH_3_NH_3_) is thermally decomposed and polymerized (at 1000°C) on graphene aerogels, leading to the formation of a-BNGA (Fig. 1). This system shows outstanding superelasticity under compression strain (≤99%) and excellent fatigue resistance (>1000 cycles) within a very broad temperature range (spanning from –196°C to ∼500°C). The chemically bonded 2D structures in a-BNGA enhance the overall aerogel integrity, thereby preventing fractures and structural damage. The analysis of thermal properties returns outstanding thermal stability under high-temperature conditions (400°C in air, 1000°C in vacuum). Moreover, the results show a thermal conductivity equal to 1.57 mW m^−1^ K^−1^ at near room temperature (<100°C), being the lowest value recorded in vacuum among solid materials to date. Such an exceptional performance is due to the ‘encasing effect’ of amorphous boron nitride over the graphene layer, whose conduction paths are reduced and phonon transport is hindered because of an enhanced scattering at the boron nitride/graphene interface. Finally, the ultralight, superelastic and thermally superinsulating a-BNGAs were tested for possible deep-space applications. The interior environment of a lunar base model wrapped with such a system experiences a longer (10-h) heating–cooling cycle compared with pristine boron nitride, graphene and silica aerogels, demonstrating the superior performance of a-BNGA in suppressing thermal radiation and heat conduction.

The promising results achieved by combining different 2D materials suggest an opportunity for further material engineering to improve the aerogel performance, since hundreds of 2D materials are available and some of them show superior thermal properties [7]. For example, MXenes offer a wide range of physicochemical properties, including absorption of infrared radiation [8]. Ti_3_C_2_T*_x_* MXene shows extremely low emissivity in the infrared range [9] and can challenge reduced graphene oxide in mechanical strength. The presence of infrared absorbers within the aerogels could reduce the radiative thermal transport, which is the dominating mechanism at low temperatures in a vacuum. Using MXene as a low-emissivity coating, an addition to or a substitute for graphene may not only decrease heat loss but add additional benefits as well, such as protection from electromagnetic interference.

This novel strategy represents a great advance in the field, paving the way toward the production of hybrid 3D aerogels made of 2D materials showing superelasticity and ultralow thermal conductivity for a variety of applications, such as extraterrestrial exploration. However, the use of mechanically robust aerogels in industrial applications at elevated temperatures, when elastic polymer foams cannot work, or at extremely low temperatures in the Arctic and Antarctic, is even more promising. Those terrestrial applications may also provide testing opportunities under practical use conditions that will ensure the reliability of aerogels in future space applications.
